# Cancer Care during the COVID-19 Pandemic: Challenges and Adaptations

**DOI:** 10.3390/curroncol30010004

**Published:** 2022-12-20

**Authors:** Shahid Ahmed

**Affiliations:** Division of Oncology, University of Saskatchewan, Medical Director Academic Saskatchewan Cancer Agency, Saskatoon, SK S7N4H4, Canada; shahid.ahmed@saskcancer.ca; Tel.: +1-3066552710; Fax: +1-3066550633

The COVID-19 pandemic is an unprecedented event that has had both acute and long-lasting effects on cancer care. It is caused by a novel virus, severe acute respiratory syndrome corona virus-2 (SARS-CoV-2), transmitted from an animal to humans [1]. As of writing this editorial, COVID-19 has infected more than 620 million people and has taken the lives of more than 6.5 million people worldwide [2]. When the World Health Organization declared the COVID-19 pandemic in March 2020, there were major concerns about the safety of cancer patients. Due to the immunosuppressive effect of the underlying cancer and cancer-related treatments, including chemotherapy, radiation, and surgery, patients with active cancer were thought to be more susceptible to COVID-19 infection and, thereby, the development of severe disease compared to the general population. During the first wave of the pandemic, a high risk of severe COVID-19 infection, and mortality in cancer patients was reported in early retrospective studies and subsequently confirmed by larger prospective or population-based studies [3,4,5,6,7]. For example, the US COVID-19 and Cancer Consortium (CCC19) prospectively examined data from 928 patients with a current or past history of cancer with COVID-19 infection and reported an all-cause mortality within 30 days of COVID-19 diagnosis of 13% [6]. It was recognized early on that primary prevention with a vaccine would be the most effective intervention to prevent COVID-19 infection and its complications in cancer patients. Through global collaborative efforts, multiple COVID-19 vaccines, including messenger RNA (mRNA) and adenovirus-vectored vaccines, have been developed and examined in clinical trials and are effective at reducing serious illness and mortality [8]. Although cancer patients undergoing active treatment were not included in the vaccine trials, evidence indicates that COVID-19 vaccination is safe and effective in cancer patients [8]. In parallel, various effective agents have been developed to reduce the severity of COVID-19 infection and hospital admission [9]. All in all, these measures, particularly COVID-19 vaccines, have helped to reduce the burden of severe illness on the healthcare system and hospital admission and have played a major role in providing cancer care.

In order to maintain sustained cancer care during this challenging time, the efficient use of available resources, the prioritization of treatments according to the magnitude of benefits in reduced capacity and compromised safety settings, and minimizing the risk of infection transmission in cancer patients and staff have become key priorities in cancer care. Several measures have been taken by the authorities involved in cancer care to eliminate or minimize the exposure of cancer patients to COVID-19-positive cases, including but were not limited to, the screening of patients and staff for COVID-19-related symptoms, minimizing patients’ visits or admission to a healthcare facility, the implementation of telemedicine services and virtual care, delaying follow-up visits for patients who had completed treatment, alternative regimens and schedules and minimizing follow-up scans to reduce visits to healthcare facilities, switching to oral or subcutaneous drugs over intravenous medicine where feasible, “treatment holidays”, “stop and go”, or oral maintenance therapy for patients with palliative treatment; increasing the use of supportive medicine (growth factors or prophylactic antibiotic) or dose modification to reduce treatment toxicities, and shorter/accelerated or hypo-fractionated radiation where appropriate. However, as we are now entering year 4 of the pandemic, it has become clear that although many measures have been taken to avoid disruptions in cancer services, the COVID-19 pandemic has had a deleterious effect on the continuum of cancer care, from cancer screening, diagnosis, treatment, and survivorship care to supportive and palliative care [10].

In this Special Issue, researchers from various jurisdictions and countries have reported the adverse effects of the COVID-19 pandemic on cancer care [11,12,13,14,15,16]. Nyk et al. retrospectively examined the impact of the COVID-19 pandemic on the risk of adverse pathology in Polish patients with early-stage prostate cancer treated with radical prostatectomy [11]. They found that patients who underwent surgery later in the pandemic had a significantly higher rate of adverse pathology than patients who had surgery earlier in the pandemic (61 vs. 43%). In a multivariable analysis, a consecutive epidemic week was associated with adverse pathology in non-high-risk patients. In a community hospital setting in New York City, Jones et al. assessed the health disparities and outcomes of cancer patients during the first wave of the pandemic in early 2020 [12]. A higher proportion of patients with COVID-19 infection or severe COVID-19 infection and COVID-19-confirmed deaths were of Hispanic ethnic background compared with patients without COVID-19. Furthermore, 100% of patients who died of COVID-19 had either Medicaid or Medicare insurance. In Germany, Habermann et al. examined the characteristics of pretreatment diagnostic assessment in patients with non-small cell lung cancer who were treated with Stereotactic Body Radiation Therapy (SBRT) during the pandemic [13]. Furthermore, they assessed the influence of diagnostic assessment on outcomes. An approximate 50% increase in the number of patients treated with SBRT was noted during the pandemic period compared to the pre-pandemic period. However, the time from pretreatment assessment to SBRT was significantly longer in patients who received treatment during the COVID-19 pandemic compared to the pre-pandemic period. The patients who did not receive a pre-SBRT positron emission tomography (PET)/computed tomography (CT) scan or those who had a pre-SBRT PET/CT scan but had to wait ≥10 weeks for SBRT had poor outcomes. Mangesius et al. evaluated the impact of the COVID-19 pandemic on radiation treatment in Austria and noted a 25% decrease in radiotherapy sessions during the first wave of the pandemic, reflecting a delay in diagnosis and referral for cancer [14]. Di Lalla et al. evaluated the satisfaction of Canadian patients undergoing active radiation treatment during the COVID-19 pandemic [15]. Although their sample size was small, 91% reported satisfaction with their initial telemedicine appointment and all patients reported being satisfied with their treatment experience during the pandemic. However, 34% of the patients felt anxious about their appointments, and 9% reported treatment delays. Lastly, the Canadian Cancer Clinical Trials Network (3CTN) study examined the impact of the pandemic on academic cancer clinical trial activity, support, and operations in Canada. The results showed delayed trial activations and lower patient recruitment, with a 67.5% decrease in patient recruitment and an 81% decrease in trial site activations compared to the pre-pandemic period [16].

Taken together, these works signify that the detrimental effects of the COVID-19 pandemic on cancer care are multi-dimensional. Cancer patients are not only at high risk of COVID-19-related complications and negative effects on their physical and mental well-being but have also experienced limited opportunities to participate in clinical trials and suffered major disruptions in essential cancer services resulting in delays in presentation, adverse pathology, and inferior outcomes. It is important to learn from the growing evidence on the impact of COVID-19 on cancer care and to develop strategies to mitigate the long-term harmful effects of the pandemic on cancer care.
